# Training the next generation of psychotraumatologists: COllaborative Network for Training and EXcellence in psychoTraumatology (CONTEXT)

**DOI:** 10.1080/20008198.2017.1421001

**Published:** 2018-01-16

**Authors:** Frédérique Vallières, Philip Hyland, Jamie Murphy, Maj Hansen, Mark Shevlin, Ask Elklit, Ruth Ceannt, Cherie Armour, Nana Wiedemann, Mette Munk, Cecilie Dinesen, Geraldine O’Hare, Twylla Cunningham, Ditte Askerod, Pernille Spitz, Noeline Blackwell, Angela McCarthy, Leonie O’Dowd, Shirley Scott, Tracey Reid, Andreas Mokake, Rory Halpin, Camila Perera, Christina Gleeson, Rachel Frost, Natalie Flanagan, Kinan Aldamman, Trina Tamrakar, Maria Louison Vang, Larissa Sherwood, Áine Travers, Ida Haahr-Pedersen, Catherine Walshe, Tracey McDonagh, Rikke Holm Bramsen

**Affiliations:** ^a^ Centre for Global Health, School of Psychology, Trinity College Dublin, Dublin, Ireland; ^b^ National College of Ireland, Dublin, Ireland; ^c^ Department of Psychology, Psychology Research Institute, Ulster University, Northern Ireland; ^d^ Department of Psychology, University of Southern Denmark, Odense, Denmark; ^e^ International Federation of the Red Cross Centre for Psychosocial Support hosted by Danish Red Cross, Copenhagen, Denmark; ^f^ Probation Board of Northern Ireland, Belfast, Northern Ireland; ^g^ Danish Children’s Centres, Odense, Denmark; ^h^ Dublin Rape Crisis Centre, Dublin, Ireland; ^i^ Police Service of Northern Ireland, Belfast, Northern Ireland; ^j^ Spirasi, Dublin, Ireland

**Keywords:** Trauma, doctoral programmes, refugees and asylum seekers, first responders, gender-based violence, childhood trauma, trauma, programas de doctorado, refugiados y solicitantes de asilo, personal de respuesta en emergencias, violencia de género, trauma infantil, 创伤, 博士项目, 移民和庇护寻求者, 现场急救员, 性别暴力, 童年暴力, • Present a new Marie Skłodowska-Curie Action programme called CONTEXT: COllaborative Network for Training and EXcellence in psychoTraumatology. • Summarize 12 research projects that are taking place across three priority populations: (i) refugees and asylum seekers, (ii) first responders, and (iii) perpetrators and survivors of childhood and gender-based violence. • Detail the mentoring and training programme and describe how the research, together with the training, will contribute towards the field of psychotraumatology.

## Abstract

In this paper we present a description of the Horizon2020, Marie Skłodowska-Curie Action funded, research and training programme CONTEXT: COllaborative Network for Training and EXcellence in psychoTraumatology. The three objectives of the programme are put forward, each of which refers to a key component of the CONTEXT programme. First, we summarize the 12 individual research projects that will take place across three priority populations: (i) refugees and asylum seekers, (ii) first responders, and (iii) perpetrators and survivors of childhood and gender-based violence. Second, we detail the mentoring and training programme central to CONTEXT. Finally, we describe how the research, together with the training, will contribute towards better policy, guidelines, and practice within the field of psychotraumatology.

## Abstract (es)

En este artículo presentamos una descripción de un nuevo programa de investigación y formación, Horizon2020, con fondos de Marie Skłodowska-Curie Action, llamado CONTEXT o ‘Red coordinadora para la formación y la excelencia en psicotraumatología’. Se presentan los tres objetivos del programa y cada uno de los cuales hace referencia a un componente clave del programa CONTEXT. Primero, resumimos los doce proyectos individuales de investigación que se llevarán a cabo en tres poblaciones prioritarias: (i) refugiados y solicitantes de asilo, (ii) personal de respuesta en emergencias y (iii) perpetradores y sobrevivientes de violencia infantil y de género. En segundo lugar, detallamos el programa de tutoría y formación, eje central de CONTEXT. Finalmente, describimos cómo la investigación, junto con la formación, contribuirá a una mejor política, directrices y práctica en el campo de la psicoterapia.

## Abstract (zh)

这篇文章中我们描述了一个新的研究和训练项目 CONTEXT（COllaborative Network for Training and EXcellence in psychoTraumatology），由‘地平线2020：玛丽·居里·斯克洛多夫斯卡行动’资助。这个项目提出了三大目标，每个目标都指向 CONTEXT项目的主要成分之一。首先，我们总结了12个独立的研究项目，它们将在三个主要人群中开展：（1）难民和庇护寻求着，（2）现场急救员，（3）童年暴力和性别暴力的施害者和受害者。其次，我们仔细叙述了 CONTEXT的核心督导和训练项目。最后，我们描述了这个研究和训练项目将如何贡献于心理创伤领域更好的政策、指导手册和实践。

## 1. Introduction

The *Diagnostic and Statistical Manual of Mental Disorders (DSM)* (American Psychological Association, 2013) and the World Health Organization’s (WHO) *International Classification of Mental and Behavioural Disorders* (WHO, 1992) serve as the primary diagnostic nosologies for psychological and psychiatric clinical research across the world. The research evidence underpinning these nosologies, however, has been predominantly generated from, and therefore generalizable to, a unique subset of the world’s population. In the case of trauma- and stress-related disorders such as Posttraumatic Stress Disorder (PTSD), over 80% of extant research has been drawn exclusively from the social systems of ‘Western’ populations, predominantly North Americans and Europeans (Fodor et al., 2014). Embedded within these nosologies is the tacit assumption that individuals throughout the world display the same psychopathological responses to trauma irrespective of cultural, individual, and contextual factors (Vallières et al., 2016). A better understanding of the nature of psychological responses, across the varying contexts within which trauma occurs, thus represents an important contribution to the field of psychotraumatology, with important implications for researchers, practitioners, and policy makers alike.

It is increasingly recognized that an international, interdisciplinary, and intersectoral approach is required if we are to adequately and equitably address the complexities of mental health to better serve the growing needs of our global population (Forsman et al., 2015). Aligned to these principles is the introduction of a Horizon2020-funded, research and training programme called CONTEXT: COllaborative Network for Training and EXcellence in psychoTraumatology (www.psychotraumanetwork.com). The goal of CONTEXT is to build capacity and expertise, and foster innovative practice and social enterprise, in the area of psychotraumatology. To achieve this goal, CONTEXT puts forward the following three interdependent objectives: (1) advance scientific knowledge regarding psychological responses to trauma across varying populations and contexts; (2) address the shortage of human resources for mental health by training highly qualified professionals with the skills to navigate the complex, contextually-specific aspects of trauma-related psychological distress across different countries, sectors, and disciplines; and (3) develop and translate evidence into innovation, improved social policy, and practice by promoting social innovation and entrepreneurial skills among the next generation of psychotraumatologists.

This paper details how each of these objectives will be achieved within CONTEXT. The first of these objectives will be achieved through 12 interconnected research projects, each of which is concerned with one or more of the following three priority research groups in Europe: (i) refugees and asylum seekers; (ii) first responders, including police officers, humanitarian workers, and individuals who work with children who have experienced direct trauma; and (iii) survivors and perpetrators of childhood and gender-based violence. These individual research projects, completed by 12 international doctoral researchers, combined with a state-of-the art mentoring and training programme, will contribute towards the achievement of objective 2. Finally, the current paper considers how achieving objectives 1 and 2 will contribute towards improved policy, guidelines, and practice within the field of psychotraumatology.

### 1.1. Priority research group 1: refugees and asylum seekers

In 2016, 65.6 million individuals were forcibly displaced worldwide with over one million refugees entering Europe (United Nation Higher Commission for Refugees [UNHCR], 2016). The psychological sequelae of forced displacement and migration pose essential questions for researchers, clinicians, and policy makers. Despite variations in reported prevalence rates for mental health disorders (i.e. depression, anxiety, PTSD) across refugee and asylum-seeking populations, research suggests they experience a higher prevalence of psychological distress compared to settled populations (Fazel, Wheeler, & Danesh, 2005). Negative responses to trauma may manifest during pre-migration, transit, and post-migration phases (Zimmerman, Kiss, & Hossain, 2011), with individuals being exposed to a series of mental health risk factors along the migration trajectory, including: war, torture, sexual and gender-based violence, poverty, marginalization, acculturation difficulties, and loss of social support (Porter & Haslam, 2005). CONTEXT aims to advance extant knowledge regarding displacement and trauma through four distinct research projects (Projects 1–4). Findings will build upon existing theoretical frameworks in refugee and asylum seeker psychology, and will be of direct translational benefit to assessment and training across a myriad of trauma contexts.

*Determining the feasibility of Red Cross volunteers delivering a WHO-developed scalable psychological intervention for forced migrants (Project 1*). Evidence suggests that non-specialized personnel can provide psychological care, and that their work contributes towards reducing the treatment gap between demand and availability of mental health services for refugees, asylum seekers, and internally displaced persons (Sijbrandij et al., 2017; Van Ginneken et al., 2013). Project 1 aims to assess the feasibility of one of WHO’s scalable psychological interventions (Problem Management Plus) (Dawson et al., 2015) as delivered by Red Cross staff and volunteers to forcibly displaced migrants. Feasibility will be assessed in terms of acceptability, practicality, and effectiveness.

*Measuring the psychosocial vulnerability of asylum-seeking and refugee populations (Project 2)*. Post-migration stressors can have a greater or equivalent impact on mental health, when compared with pre-migration trauma (Chen, Hall, Ling, & Renzaho, 2017; Chu, Keller, & Rasmussen, 2013; Kartal & Kiropoulos, 2016; Sonne et al., 2016). This study will develop a scalable means of identifying psychosocial vulnerability in the post-migration phase. It aims to identify the most salient post-migration factors that differentially predict mental health outcomes in asylum-seeking and refugee populations. The resultant instrument will be available to practitioners throughout the EU to assist in triaging clients from the point of initial assessment.

*Assessing the validity of Complex Posttraumatic Stress Disorder (CPTSD) among asylum-seeking and refugee populations who have experienced sexual violence (Project 3*). Knowledge regarding CPTSD, as per the proposed International Classification of Diseases 11th version (ICD-11) guidelines (Maercker et al., 2013), is typically informed by research documenting the negative sequelae of childhood trauma. There is a dearth of literature investigating CPTSD among refugee samples (Nickerson et al., 2016). This project will evaluate the validity of CPTSD proposals, and assess key etiological predictors of CPTSD among refugees and asylum seekers who have experienced sexual violence. The findings of this project will advance knowledge regarding manifestations of traumatic response across diverse cultural groups who have been exposed to sexual violence.

*Exploring the mechanisms of intergenerational trauma among refugees (Project 4*). The global mental health literature demonstrates that trauma experienced by a parental figure can adversely affect offspring and subsequent generations (Dalgaard, Todd, Daniel, & Montgomery, 2016; Sirikantraporn & Green, 2016). The impact of trauma on the dyadic relationship is further complicated when a parent has experienced torture (Daud, Skoglund, & Rydelius, 2005). This study will seek to elucidate communicative mechanisms underpinning the translation of trauma across generations. Findings will inform EU treatment programmes for individuals who have a history of torture and who have been recently reunited with their children.

### 1.2. Priority research group 2: first responders

Working with survivors of trauma often comes with a personal cost, measured in increased levels of posttraumatic stress, secondary traumatization, anxiety, depression, and burnout (Antares Foundation, 2012; Connorton, Perry, Hemenway, & Miller, 2012; Cornille & Meyers, 1999; Strohmeier & Scholte, 2015). Consequently, the provision of appropriate support should be a priority for organizations whose staff and volunteers are exposed to such experiences (IASC, 2007). Studies investigating the mental health of first responders identify common risk and protective factors that determine first responder wellbeing, including individual, interpersonal, and organizational factors (Ager et al., 2012; Hearns & Deeny, 2007; McCall & Salama, 1999; Papazoglou, 2013; Rubin et al., 2016; Thormar et al., 2014). However, conflicting findings remain regarding the specific role played by each of these factors and research often disregards the context under which these factors are assessed. For example, some studies have found that ‘years of service’ acts as a protective factor against mental health problems, while others have found that it acts as a risk factor for deleterious outcomes (Dagan, Ben-Porat, & Itzhaky, 2016; Meyers & Cornille, 2002). Therefore, while common risk and protective factors may exist, they are also intrinsically linked to, and defined by, a helpers’ environmental and cultural context, making it imperative to formulate research that is sufficiently sensitive to capture this specificity and variation (Vallières et al., 2016). CONTEXT aims to improve practice, procedures, and guidelines for organizations that are responsible for the protection and care of first responders, working in different trauma contexts, through four projects (Projects 5–8).

*Managerial practices to ensure the well-being of humanitarian volunteers in conflict situations (Project 5*). Managerial and organizational factors can significantly impact humanitarian aid workers’ wellbeing (Brooks et al., 2015; Thormar et al., 2013). Through a realist evaluation of the International Federation of Red Cross and Red Crescent Societies’ (IFRC) ‘Caring for Volunteers’ (IFRC Reference Centre for Psychosocial Support, 2012) programme across two conflict or post-conflict settings, Project 5 aims to elucidate how managerial factors can improve volunteer support structures within the IFRC, with a view to developing better policies and guidelines for volunteer care in emergency response settings.

*Police Service of Northern Ireland (PSNI) trauma risk management strategy evaluation study (Project 6)*. Police officers, on average, experience over three traumatic incidents for every six months of service, making them highly vulnerable to PTSD (Patterson, 2001). Project 6 evaluates the efficacy of post-incident debriefings in reducing secondary traumatic stress among police officers working in the Police Service of Northern Ireland. The project further aims to develop an empirically-based debriefing evaluation tool specific to the PSNI.

*Secondary traumatization in mental health professionals working with victims of child abuse (Project 7)*. Professionals working with survivors of child abuse are at high risk for adverse mental health outcomes (Walker, 2004). However, research also suggests the co-occurrence of emotional exhaustion and high job satisfaction (Stalker, Mandell, Frensch, Harvey, & Wright, 2007). Project 7 investigates the interplay of risk and protective factors for mental health outcomes among employees of a child protection service: the Danish Children Centres. Results will inform recommendations to promote mental health outcomes for those caring for traumatized youths.

*Identifying context-specific risk for discrete trauma-exposed PSNI officer populations (Project 8*). Over the last three years the number of sick days taken by PSNI officers due to poor mental health or stress has increased by more than 60%, reaching nearly 40,000 sick days last year alone (Lindsay, 2016). This study aims to identify the contextual and role-specific risk factors that predict negative mental health outcomes for PSNI officers. Previous research has often focused solely on individual domains of risk such as social support (Evans, Pistrang, & Billings, 2013). However, it is crucial to address the multifactorial nature of stress and trauma in policing, including personal, organizational, and operational risk factors (Habersaat, Geiger, Abdellaoui, & Wolf, 2015). The results of this study will be used to create context-sensitive recommendations for a trauma risk management strategy for the PSNI.

### 1.3. Priority research group 3: survivors and perpetrators of childhood and gender-based violence

Experiencing physical or sexual violence is a potent risk factor for a range of adverse physical-, mental-, and social-health outcomes (Coker et al., 2002; Felitti et al., 1998). The United Nations defines gender-based violence (GBV) as violence ‘that is directed against a woman because she is a woman or that affects women disproportionately’ (UN CEDAW Committee, 1992). Closely linked to GBV is violence against children (VAC). The two forms of violence frequently overlap and co-occur, sharing many risk factors and consequences (Guedes, Bott, Garcia-Moreno, & Colombini, 2016). VAC and GBV are major public health and human rights concerns globally; official prevalence statistics, although worryingly high, are likely underestimated due to chronic under-reporting (Watts & Zimmerman, 2002).

Evidence-based strategies and sound policies to address risk, support survivors, and rehabilitate perpetrators are crucial. CONTEXT projects 9–12 focus on survivors and perpetrators of VAC and GBV, and aim to investigate complex interactions of risk factors for experiencing or perpetrating violence and identify targets for intervention. Projects 9–12 also aim to describe facilitators of recovery for survivors and protective factors that have potential to enhance the likelihood of perpetrators’ rehabilitation. Central to the analyses is an attentiveness to the ways in which traumatic experiences can interact with other factors (e.g. social stigmas and inequalities, socio-economic and family status, sex, gender identity, age, pre-existing health and mental health status) to compound one’s likelihood of experiencing violence, to perpetrate it, to recover from it, or to be rehabilitated. Projects are designed to produce recommendations for one of three partner organizations: the Probation Board of Northern Ireland (PBNI), the Danish Children’s Centres, and the Dublin Rape Crisis Centre (DRCC). However, it is hoped that the findings will also contribute to trauma-informed approaches to combatting GBV and VAC with generalizability across contexts.

*Investigating Childhood Trauma and Mental Health in an Offender Population (Project 9)*. While not all people who are maltreated as children become violent perpetrators in later life, experiencing abuse has been identified as a risk factor for criminality (Ardino, 2012; DeLisi, Kosloski, Vaughn, Caudill, & Trulson, 2014). Using a randomly selected sample of case file data (*n* = 120,000) from the PBNI, project 9 will examine associations between early trauma, mental health problems, and violent offending. Findings will inform recommendations for preventive intervention.

*Investigating Polyvictimization in Child Abuse Cases (Project 10*). This examines multiple victimizations (polyvictimization; Finkelhor, Ormrod, & Turner, 2007) within a multidisciplinary child protection setting: Danish Children’s Centres. Profiles of polyvictimization and their associations with adverse outcomes will be investigated. Findings will inform case planning and interventions for polyvictimized children.

*Gender-Specific Facilitators and Barriers to Accessing Rape and Sexual Assault Services (Project 11*). The experience of sexual violence can have profound psychological effects on an individual. Despite this, few survivors seek professional help (Parcesepe, Martin, Pollock, & García-Moreno, 2015; Ullman, 2007). This project will identify barriers and facilitators to seeking and accessing help in the wake of a sexual assault. Results will inform recommendations to restructure existing services and awareness campaigns of a sexual assault support service in the Republic of Ireland, in collaboration with the Dublin Rape Crisis Centre.

*Developmental Psychosocial and Trauma-related Factors Influencing Offender Desistance (Project 12*). Understanding the process whereby offenders desist from violent offending is imperative to reduce the burden of recidivism and to prevent the cycle of trauma from progressing. Psychological factors have been particularly under-investigated. This project, conducted with the PBNI, will identify both risk and protective factors that will inform strategies aimed at reducing the risk of reoffending.

## 2. Training

Aligned to the WHO’s *Policies and Practices for Mental Health in Europe* and the EU’s *Joint Action on Mental Health and Wellbeing* (Forsman et al., 2015), CONTEXT emphasizes interdisciplinary approaches that go beyond traditional training for psychotraumatologists. Whereas common psychotraumatology training typically occurs in an academic setting, or as a non-academic clinical subspecialist qualification, CONTEXT’s training brings these elements together, to provide a framework for best-practice for psychotraumatology training that can be adopted across Europe.

The training provided by CONTEXT is intended to equip a new generation of psychotraumatologists with a portfolio of expertise and leadership skills needed to work within the humanitarian, public-service, policy, government, non-governmental, and academic sectors by promoting: (1) international networking and intersectoral collaboration by means of fellows spending at least 50% of their time in the non-academic settings; (2) interdisciplinary training by means of formal taught programmes delivered by both academic and non-academic experts from diverse backgrounds; and (3) experiencing and learning how to conduct research across academic and non-academic settings, so as to ensure that empirical findings can be efficiently translated into clinical and operational practice, ultimately improving the lives of traumatized persons and those that work with victims of trauma. The result is a unique research training designed to increase human resources for mental health in Europe. Upon completion, graduates will be in a unique position to convert their research findings into interventions and practices for social benefit, thereby contributing towards closing the existing gap between research and practice sectors.

## 3. Implications for science, policy, and practice

Mental health disorders make up a significant proportion of Europe’s overall burden of disease (Wittchen et al., 2011) and result in significant annual economic costs to the continent (Olesen, Gustavsson, Svensson, Wittchen, & Jonsson, 2012). The CONTEXT consortium, with its emphasis on an international, interdisciplinary, and intersectoral approach, will contribute towards addressing the complexities of trauma, to better serve the changing and growing mental health needs of the European population (Forsman et al., 2015). Specifically, and aligned to the three recommendations outlined in the Roadmap for Mental Health Research in Europe Project, CONTEXT will: (1) advance scientific understandings of the causes, risk-, and protective-factors for mental health; (2) improve the capacity and availability of human resources for mental health to implement mental health interventions; to ultimately (3) reduce disparities in mental health across various contexts and cultures. These recommendations will be achieved through Europe’s longstanding tradition (Turner, 2013) of conducting innovative science in the field of psychotraumatology: using approaches that recognize the importance of the context-specific nature within which trauma can occur, and how this influences trauma response and trauma recovery.

## References

[CIT0001] Ager A., Pasha E., Yu G., Duke T., Eriksson C., & Cardozo B. L. (2012). Stress, Mental Health, and Burnout in National Humanitarian Aid Workers in Gulu, Northern Uganda. *Journal of Traumatic Stress*, 25(6), 713–7. 23225036 10.1002/jts.21764

[CIT0002] American Psychological Association (2013). *Diagnostic and statistical manual of mental disorders* (5th ed.). Washington, DC: Author.

[CIT0003] Antares Foundation (2012). *Managing stress in humanitarian workers* . Retrieved from https://www.antaresfoundation.org/FileLibrary/file6782.pdf

[CIT0004] Ardino V. (2012). Offending behaviour: The role of trauma and PTSD. *European Journal of Psychotraumatology*, 3(1), 18968. 10.3402/ejpt.v3i0.18968 PMC3402156 22893844

[CIT0005] Brooks S. K., Dunn R., Sage C. A., Amlot R., Greenberg N., & Rubin G. J. (2015). Risk and resilience factors affecting the psychological wellbeing of individuals deployed in humanitarian relief roles after a disaster. *Journal of Mental Health*, 24(6), 385–413. 26452755 10.3109/09638237.2015.1057334

[CIT0006] Chen W., Hall B. J., Ling L., & Renzaho A. M. (2017). Pre-migration and post-migration factors associated with mental health in humanitarian migrants in Australia and the moderation effect of post-migration stressors: Findings from the first wave data of the BNLA cohort study. *Lancet Psychiatry*, 4(3), 218–229. 28161455 10.1016/S2215-0366(17)30032-9

[CIT0007] Chu T., Keller A. S., & Rasmussen A. (2013). Effects of post-migration factors on PTSD outcomes among immigrant survivors of political violence. *Journal of Immigrant Minority Health*, 15(5), 890–897. 22976794 10.1007/s10903-012-9696-1

[CIT0008] Coker A. L., Davis K. E., Arias I., Desai S., Sanderson M., Brandt H. M., & Smith P. H. (2002). Physical and mental health effects of intimate partner violence for men and women. *American Journal of Preventive Medicine*, 23(4), 260–268. 12406480 10.1016/s0749-3797(02)00514-7

[CIT0009] Connorton E., Perry M. J., Hemenway D., & Miller M. (2012). Humanitarian relief workers and trauma-related mental illness. *Epidemiologic Reviews*, 34(1), 145–155. 22180469 10.1093/epirev/mxr026

[CIT0010] Cornille T. A., & Meyers T. W. (1999). Secondary traumatic stress among child protective service workers: Prevalence, severity and predictive factors. *Traumatology*, 5(1). doi:10.1177/153476569900500105

[CIT0011] Dagan S. W., Ben-Porat A., & Itzhaky H. (2016). Child protection workers dealing with child abuse: The contribution of personal, social and organizational resources to secondary traumatization. *Child Abuse & Neglect*, 51, 203–211. 26549769 10.1016/j.chiabu.2015.10.008

[CIT0012] Dalgaard N. T., Todd B. K., Daniel S. I. F., & Montgomery E. (2016). The transmission of trauma in refugee families: Associations between intra-family trauma communication style, children’s attachment security and psychosocial adjustment. *Attachment & Human Development*, 18(1), 69–89. 26608277 10.1080/14616734.2015.1113305

[CIT0013] Daud A., Skoglund E., & Rydelius P. A. (2005). Children in families of torture victims: Transgenerational transmission of parents’ traumatic experiences to their children. *International Journal of Social Welfare*, 14(1), 23–32.

[CIT0014] Dawson K. S., Bryant R. A., Harper M., Kuowei Tay A., Rahman A., Schafer A., & Van Ommeren M. (2015). Problem Management Plus (PM+): A WHO transdiagnostic psychological intervention for common mental health problems. *World Psychiatry*, 14(3), 354–357. 26407793 10.1002/wps.20255 PMC4592660

[CIT0015] DeLisi M., Kosloski A. E., Vaughn M. G., Caudill J. W., & Trulson C. R. (2014). Does childhood sexual abuse victimization translate into juvenile sexual offending? New evidence. *Violence and Victims*, 29(4), 620–635. 25199390 10.1891/0886-6708.vv-d-13-00003

[CIT0016] Evans R., Pistrang N., & Billings J. (2013). Police officers’ experiences of supportive and unsupportive social interactions following traumatic incidents. *European Journal of Psychotraumatology*, 4(1), 19696. 10.3402/ejpt.v4i0.19696 PMC3600426 23516046

[CIT0017] Fazel M., Wheeler J., & Danesh J. (2005). Prevalence of serious mental disorder in 7000 refugees resettled in western countries: A systematic review. *Lancet*, 365(9467), 1309–1314. 15823380 10.1016/S0140-6736(05)61027-6

[CIT0018] Felitti V. J., Anda R. F., Nordenberg D., Williamson D. F., Spitz A. M., Edwards V., … Marks J. S. (1998). Relationship of childhood abuse and household dysfunction to many of the leading causes of death in adults. The Adverse Childhood Experiences (ACE) Study. *American Journal of Preventive Medicine*, 14(4), 245–258. 9635069 10.1016/s0749-3797(98)00017-8

[CIT0019] Finkelhor D., Ormrod R. K., & Turner H. A. (2007). Poly-victimization: A neglected component in child victimization. *Child Abuse and Neglect*, 31(1), 7–26. 17224181 10.1016/j.chiabu.2006.06.008

[CIT0020] Fodor K. E., Unterhitzenberger J., Chou C. Y., Kartal D., Leistner S., Milosavljevic M., … Alisic E. (2014). Is traumatic stress research global? A bibliometric analysis. *European Journal of Psychotraumatology*, 5, 23269.

[CIT0021] Forsman A. K., Wahlbeck K., Aaro L. E., Alonso J., Barry M. M., Brunn M., … Varnik A. (2015). Research priorities for public mental health in Europe: Recommendations of the ROAMER project. *European Journal of Public Health*, 25(2), 249–254. 25678606 10.1093/eurpub/cku232

[CIT0022] Guedes A., Bott S., Garcia-Moreno C., & Colombini M. (2016). Bridging the gaps: A global review of intersections of violence against women and violence against children. *Global Health Action*, 9, 31516. 27329936 10.3402/gha.v9.31516 PMC4916258

[CIT0023] Habersaat S. A., Geiger A. M., Abdellaoui S., & Wolf J. M. (2015). Health in police officers: Role of risk factor clusters and police divisions. *Social Science and Medicine*, 143(Supplement C), 213–222. 26364008 10.1016/j.socscimed.2015.08.043 PMC4601933

[CIT0024] Hearns A., & Deeny P. (2007). The value of support for aid workers in complex emergencies: A phenomenological study. *Disaster Management & Response*, 5(2), 28–35. 17517360 10.1016/j.dmr.2007.03.003

[CIT0025] IASC (2007). *The inter-agency standing committee guidelines on mental health and psychosocial support in emergency settings*. Geneva: IASC. 10.1080/09540261.2022.2147420 36502397

[CIT0026] IFRC Reference Centre for Psychosocial Support (2012). *Caring for Volunteers: A psychosocial support toolkit* . Retrieved from http://pscentre.org/wp-content/uploads/volunteers_EN.pdf

[CIT0027] Kartal D., & Kiropoulos L. (2016). Effects of acculturative stress on PTSD, depressive, and anxiety symptoms among refugees resettled in Australia and Austria. *European Journal of Psychotraumatology*, 7, 28711. 10.3402/ejpt.v7.28711 PMC4756629 26886488

[CIT0028] Lindsay M. (2016, 6). A uniform is the only difference. In *Speech given at the annual Police Federation of Northern Ireland Conference* Belfast, Northern Ireland.

[CIT0029] Maercker A., Brewin C. R., Bryant R. A., Cloitre M., Van Ommeren M., Jones L. M., … Reed G. M. (2013). Diagnosis and classification of disorders specifically associated with stress: Proposals for ICD-11. *World Psychiatry*, 12(3), 198–206. 24096776 10.1002/wps.20057 PMC3799241

[CIT0030] McCall M., & Salama P. (1999). Selection, training, and support of relief workers: An occupational health issue. *BMJ*, 318(7176), 113–116. 9880288 10.1136/bmj.318.7176.113 PMC1114577

[CIT0031] Meyers T. W., & Cornille A. T. (2002). The trauma of working with traumatized children In Figley C. (Ed.), *Treating compassion fatigue*. New York: Brunner-Routledge.

[CIT0032] Nickerson A., Cloitre M., Bryant R. A., Schnyder U., Morina N., & Schick M. (2016). The factor structure of complex posttraumatic stress disorder in traumatized refugees. *European Journal of Psychotraumatology*, 7, 33253. 10.3402/ejpt.v7.33253 PMC5165057 27989268

[CIT0033] Olesen J., Gustavsson A., Svensson M., Wittchen H.-U., & Jönsson B. (2012). The economic cost of brain disorders in Europe. *European Journal of Neurology*, 19(1), 155–162. 22175760 10.1111/j.1468-1331.2011.03590.x

[CIT0034] Papazoglou K. (2013). Conceptualizing Police Complex Spiral Trauma and its applications in the police field. *Traumatology*, 19(3), 196–209.

[CIT0035] Parcesepe A. M., Martin S. L., Pollock M. D., & García-Moreno C. (2015). The effectiveness of mental health interventions for adult female survivors of sexual assault: A systematic review. *Aggression and Violent Behavior*, 25(Part A), 15–25.

[CIT0036] Patterson G. T. (2001). The relationship between demographic variables and exposure to traumatic incidents among police officers. *Australasian Journal of Disaster and Trauma Studies*, 5(2). Available from: http://trauma.massey.ac.nz/issues/2001-2/patterson2.htm

[CIT0037] Porter M., & Haslam N. (2005). Predisplacement and postdisplacement factors associated with mental health of refugees and internally displaced persons: A meta-analysis. *JAMA*, 294(5), 602–612. 16077055 10.1001/jama.294.5.602

[CIT0038] Rubin G. J., Harper S., Williams P. D., Öström S., Bredbere S., Amlôt R., & Greenberg N. (2016). How to support staff deploying on overseas humanitarian work: A qualitative analysis of responder views about the 2014/15 West African Ebola outbreak. *European Journal of Psychotraumatology*, 7, 30933. 27863536 10.3402/ejpt.v7.30933 PMC5116059

[CIT0039] Sijbrandij M., Acarturk C., Bird M., Bryant R. A., Burchert S., Carswell K., … Cuijpers P. (2017). Strengthening mental health care systems for Syrian refugees in Europe and the Middle East: Integrating scalable psychological interventions in eight countries. *European Journal of Psychotraumatology*, 8(sup2), 1388102. 29163867 10.1080/20008198.2017.1388102 PMC5687806

[CIT0040] Sirikantraporn S., & Green J. (2016). Introduction: Multicultural Perspectives of Intergenerational Transmission of Trauma. *Journal of Aggression, Maltreatment & Trauma*, 25(6), 559–560.

[CIT0041] Sonne C., Carlsson J., Bech P., Vindbjerg E., Mortensen E. L., & Elklit A. (2016). Psychosocial predictors of treatment outcome for trauma-affected refugees. *European Journal of Psychotraumatology*, 7, 30907. 27251179 10.3402/ejpt.v7.30907 PMC4889772

[CIT0042] Stalker C. A., Mandell D., Frensch K. M., Harvey C., & Wright M. (2007). Child welfare workers who are exhausted yet satisfied with their jobs: How do they do it? *Child & Family Social Work*, 12(2), 182–191.

[CIT0043] Strohmeier H., & Scholte W. F. (2015). Trauma-related mental health problems among national humanitarian staff: A systematic review of the literature. *European Journal of Psychotraumatology*, 6, 28541. 26589256 10.3402/ejpt.v6.28541 PMC4654769

[CIT0044] Thormar S. B., Gersons B. P., Juen B., Djakababa M. N., Karlsson T., & Olff M. (2013). Organizational factors and mental health in community volunteers. The role of exposure, preparation, training, tasks assigned, and support. *Anxiety Stress Coping*, 26(6), 624–642. 23205850 10.1080/10615806.2012.743021

[CIT0045] Thormar S. B., Gersons B. P. R., Juen B., Djakababa M. N., Karlsson T., & Olff M. (2014). The impact of disaster work on community volunteers: The role of peri-traumatic distress, level of personal affectedness, sleep quality and resource loss, on post-traumatic stress disorder symptoms and subjective health. *Journal of Anxiety Disorders*, 28(8), 971–977. 25445088 10.1016/j.janxdis.2014.10.006

[CIT0046] Turner S. (2013). Psychotraumatology in Europe: A personal history. *European Journal of Psychotraumatology*, 4(1), 21305. 10.3402/ejpt.v4i0.21305 PMC3676534 23755329

[CIT0047] UN CEDAW Committee (1992, 1 29). *Article 6, General Recommendation No. 19 on Violence against women*. Adopted at the 11th session, 1992, A/47/38. UN General Assembly, Geneva.

[CIT0048] Ullman S. E. (2007). Mental health services seeking in sexual assault victims. *Women & Therapy*, 30(1–2), 61–84.

[CIT0049] United Nation Higher Commission for Refugees [UNHCR] (2016). Figures at a glance. Retrieved from http://www.unhcr.org/en-ie/figures-at-a-glance.html

[CIT0050] Vallières F., Ceannt R., Hyland P., Bramsen R., Hansen M., & Murphy J. (2016). The need to contextualise psychotraumatology research. *The Lancet Global Health*, 4(2), e87–e88. 26823221 10.1016/S2214-109X(15)00244-2

[CIT0051] Van Ginneken N., Tharyan P., Lewin S., Rao G. N., Meera S. M., Pian J., … Patel V. (2013). Non-specialist health worker interventions for the care of mental, neurological and substance-abuse disorders in low- and middle-income countries. *Cochrane Database Systematic Reviews*, 11, Cd009149. 10.1002/14651858.CD009149.pub2 24249541

[CIT0052] Walker M. (2004). Supervising practitioners working with survivors of childhood abuse: Counter transference; secondary traumatization and terror. *Psychodynamic Practice*, 10(2), 173–193.

[CIT0053] Watts C., & Zimmerman C. (2002). Violence against women: Global scope and magnitude. *Lancet*, 359(9313), 1232–1237. 11955557 10.1016/S0140-6736(02)08221-1

[CIT0054] WHO (1992). *The ICD-10 classification of mental and behavioural disorders: Clinical descriptions and diagnostic guidelines* (10th ed.). Geneva: Author.

[CIT0055] Wittchen H. U., Jacobi F., Rehm J., Gustavsson A., Svensson M., Jönsson B., … Steinhausen H. C. (2011). The size and burden of mental disorders and other disorders of the brain in Europe 2010. *European Neuropsychopharmacology*, 21(9), 655–679. 21896369 10.1016/j.euroneuro.2011.07.018

[CIT0056] Zimmerman C., Kiss L., & Hossain M. (2011). Migration and health: A framework for 21st century policy-making. *PLoS Medicine*, 8(5), e1001034. 21629681 10.1371/journal.pmed.1001034 PMC3101201

